# Association of *Bordetella *dermonecrotic toxin with the extracellular matrix

**DOI:** 10.1186/1471-2180-10-247

**Published:** 2010-09-25

**Authors:** Aya Fukui-Miyazaki, Shigeki Kamitani, Masami Miyake, Yasuhiko Horiguchi

**Affiliations:** 1 Department of Molecular Bacteriology, Research Institute for Microbial Diseases, Osaka University, Osaka, Japan; 2 Laboratory of Veterinary Public Health, Department of Veterinary Environmental Sciences, Osaka Prefecture University, Osaka, Japan

## Abstract

**Background:**

*Bordetella *dermonecrotic toxin (DNT) causes the turbinate atrophy in swine atrophic rhinitis, caused by a *Bordetella bronchiseptica *infection of pigs, by inhibiting osteoblastic differentiation. The toxin is not actively secreted from the bacteria, and is presumed to be present in only small amounts in infected areas. How such small amounts can affect target tissues is unknown.

**Results:**

Fluorescence microscopy revealed that DNT associated with a fibrillar structure developed on cultured cells. A cellular component cross-linked with DNT conjugated with a cross-linker was identified as fibronectin by mass spectrometry. Colocalization of the fibronectin network on the cells with DNT was also observed by fluorescence microscope. Several lines of evidence suggested that DNT interacts with fibronectin not directly, but through another cellular component that remains to be identified. The colocalization was observed in not only DNT-sensitive cells but also insensitive cells, indicating that the fibronectin network neither serves as a receptor for the toxin nor is involved in the intoxicating procedures. The fibronectin network-associated toxin was easily liberated when the concentration of toxin in the local environment decreased, and was still active.

**Conclusions:**

Components in the extracellular matrix are known to regulate activities of various growth factors by binding and liberating them in response to alterations in the extracellular environment. Similarly, the fibronectin-based extracellular matrix may function as a temporary storage system for DNT, enabling small amounts of the toxin to efficiently affect target tissues or cells.

## Background

Pathogenic bacteria of the genus *Bordetella *produce dermonecrotic toxin (DNT), which activates Rho GTPases through its transglutaminase activity resulting in deamidation or polyamination [1-3]. DNT is a single chain polypeptide of 1,464 amino acids, with an N-terminal region of at least 54 amino acids responsible for binding to a receptor on target cells [4] and a C-terminal region of about 300 amino acids conferring the transglutaminase activity [5]. The receptor for DNT is still unknown. The activated Rho GTPases cause aberrant Rho-dependent phenotypes [6,7], which likely lead to some of the pathological changes observed during *Bordetella *infections. For example, the turbinate atrophy in atrophic rhinitis, a *Bordetella *infection of pigs, is caused by DNT acting on osteoblastic cells [8-13]. However, there has been no evidence that DNT is actively secreted from the bacteria, and less than 0.75% (0.60 ng/10^9 ^CFU) of produced DNT was detected in culture supernatant of *B. bronchiseptica *and *B. pertussis *(unpublished data). It is unknown how this small amount of DNT exerts toxicity against target cells such as osteoblasts covered by epithelial cells and connective tissue.

While attempting to identify the receptor for DNT, we found that DNT associated temporarily with fibronectin (FN)-based extracellular matrix (ECM), on both DNT-sensitive and insensitive cells, indicating that the FN network does not serve as a functional receptor for DNT. We hypothesized that the FN network functions as a temporary storage system for DNT, enabling the small amount of the toxin to effectively reach target cells across the epithelia and connective tissue.

## Results

### DNT binds to the FN-based ECM network

**Figure 1:**
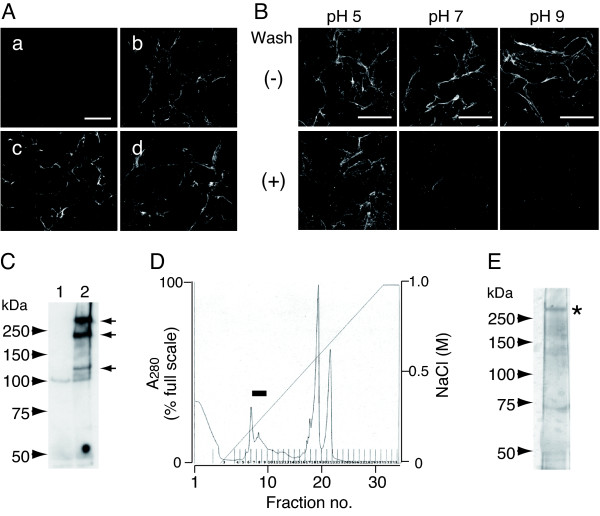
**DNT is associated with the fibrillar structure on MC3T3-E1 cells**. **(A) **The cells were treated with DNT (a and b), 5-FAM-DNT (c), or SBED-DNT (d) as mentioned in Methods. The cells were stained without wash as follows. DNT was detected with a combination of anti-DNT polyclonal antibody and Alexa 488-conjugated secondary antibody (b). The DNT-treated cells were stained with only the secondary antibody for the control (a). 5-FAM-DNT was visualized with direct fluorescence microscopy (c). SBED-DNT was detected with Alexa 488-conjugated streptavidin (d). Note that the association of DNT with the fibrillar structure was observed independently of the detection method. Bar, 5 μm. **(B) **MC3T3-E1 cells were incubated with DNT at different pH and stained with anti-DNT polyclonal antibody. The cells were washed once (lower panels) or not washed (upper panels) before fixation. Bar, 5 μm. **(C) **Cellular components cross-linked by SBED-DNT. MC3T3-E1 cells were incubated with (lane 2) or without (lane 1) SBED-DNT. After the cross-linking procedure, the insoluble fraction was prepared as described in Methods and subjected to SDS-PAGE with a 6% acrylamide gel containing 6 M urea under reducing conditions. Cellular components labeled by biotin through SBED were detected by Western blotting with HRP-conjugated streptavidin. Arrows indicate cellular components cross-linked with SBED-DNT. **(D) **Mini Q column chromatographic profile of the insoluble fraction of MC3T3-E1 cells treated and cross-linked with SBED-DNT. The cellular component with the higher molecular weight was eluted in fractions 6 to 8 (bold bar). **(E) **SDS-PAGE of fraction 7. The cellular component with the higher molecular weight is indicated with an asterisk.

**Figure 2:**
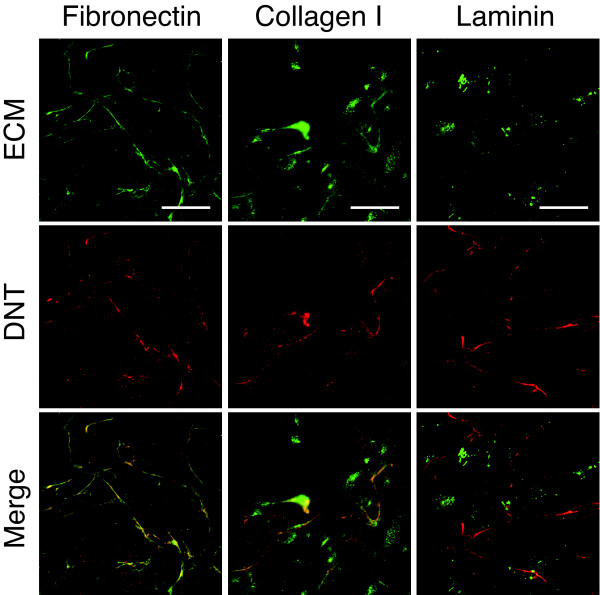
**Colocalization of DNT with the ECM components**. MC3T3-E1 cells incubated with DNT were stained with anti-DNT monoclonal antibody or polyclonal antibody against FN, collagen type I or laminin. Bars, 5 μm.

While attempting to identify a receptor for DNT, we found that DNT was distributed along with a fibrillar structure on the surface of MC3T3-E1 cells (Fig. 1A), suggesting an affinity for some component of the ECM. This affinity appeared to be dependent on pH: most of the bound toxin was easily washed away from the cell surface at pH 7 or 9, whereas a detectable amount of DNT remained bound after washing at pH 5 (Fig. 1B). To identify the cellular component associated with DNT, we tried to cross-link the toxin with its counterpart using Sulfo-SBED, a trifunctional cross-linker, which labels the cross-linked molecule with biotin. Sulfo-SBED-labeled DNT (SBED-DNT), which had a similar distribution to the native toxin (Fig. 1A-d), transferred biotin to at least three distinct cellular components in NP-40 insoluble fraction detected by Western blotting (Fig. 1C). Only the component with the highest molecular weight could be isolated by anion-exchange chromatography (Fig. 1D and 1E), and identified as mouse FN by mass spectrometry. FN is a major component organizing the ECM. We examined if the toxin colocalizes with the FN network by staining FN or other ECM components, such as collagen type I and laminin. DNT was found to be well colocalized with the FN network and partly colocalized with the collagen type I, but not colocalized with laminin (Fig. 2).

**Figure 3:**
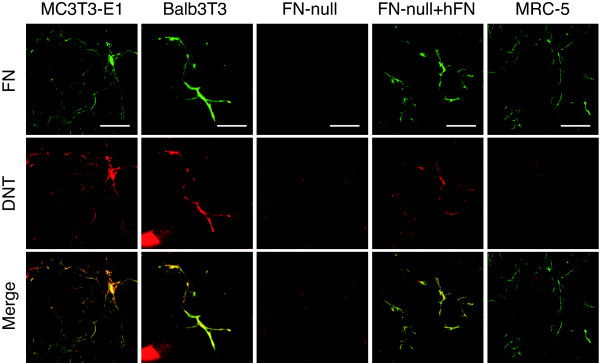
**Colocalization of DNT with the FN network on various cells**. Cells were incubated with DNT and stained with anti-DNT monoclonal antibody and anti-FN polyclonal antibody. FN-null cells were incubated with or without human FN (hFN) before DNT treatment. Bars, 5 μm.

**Figure 4:**
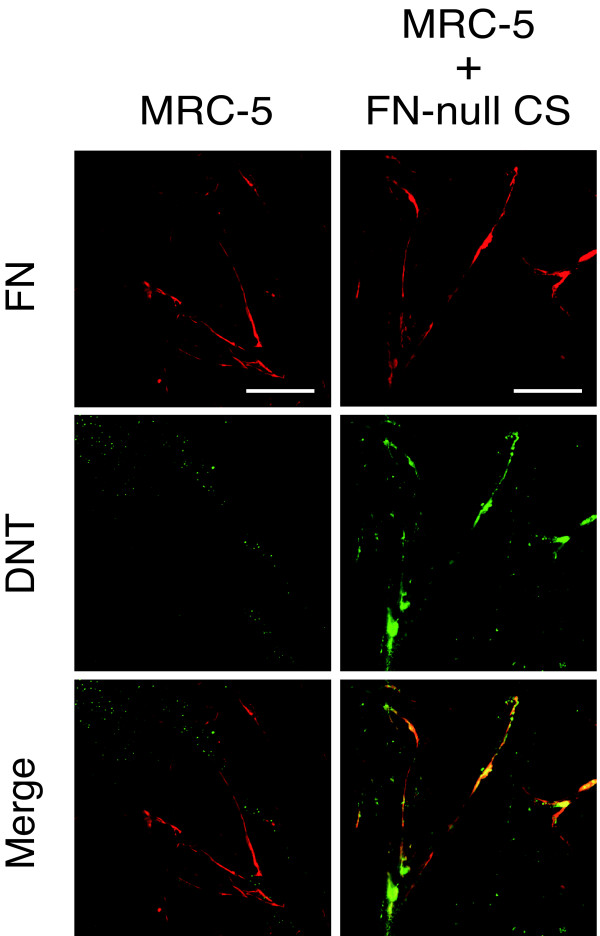
**Colocalization of DNT with the FN network on MRC-5 cells supplemented with the culture supernatant of FN-null cells**. MRC-5 cells, which were pre-cultured with or without the culture supernatant of FN-null cells (FN-null CS), were incubated with DNT and stained with anti-DNT monoclonal antibody and anti-FN polyclonal antibody. Bars, 5 μm.

Besides MC3T3-E1 cells, which are sensitive to DNT, DNT-insensitive Balb3T3 cells also showed the colocalization of DNT with the FN network (Fig. 3). FN-null cells, which are fibroblastic cells isolated from FN knock-out mouse embryos and is sensitive to DNT, did not show a fibrillar distribution of the toxin (Fig. 3). These result indicate that the association of DNT with FN is not related to the intoxication. When human FN was supplied to the culture, FN-null cells showed the colocalization of the toxin and FN. In contrast, DNT did not colocalize with the FN network developed on MRC-5 cells (Fig. 3). These results suggest that DNT does not interact directly with FN, and another cellular component, which is present in the culture of FN-null cells but not MRC-5 cells, is necessary for the interaction. In fact, MRC-5 cells supplemented with the culture supernatant of FN-null cells showed the colocalization of DNT and the FN network (Fig. 4). Treatment with heat at 95°C or proteinase K abolished the ability of the culture supernatant to recruit DNT to the FN network, which indicates that the unknown component exists in the culture supernatant of FN-null cells and contains a protein moiety (data not shown).

### Screening for a molecule mediating the colocalization of DNT and the FN network

**Figure 5:**
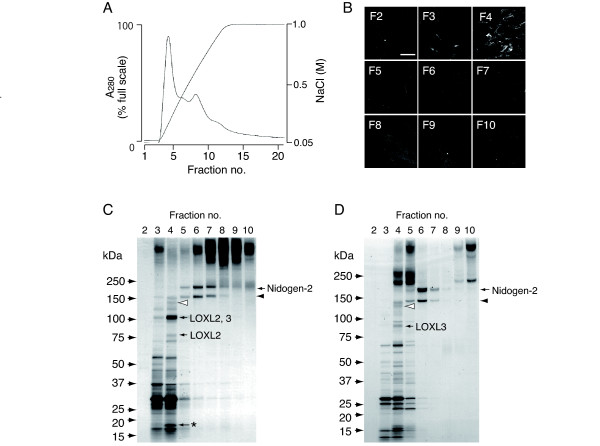
**Screening for a molecule mediating the association of DNT with the FN network**. **(A) **Profile of Mono Q anion-exchange chromatography of the culture supernatant of FN-null cells. **(B) **The association of DNT with the FN network of MRC-5 cells supplemented with each fraction from the chromatography. MRC-5 cells seeded in a 24-well plate were incubated overnight with eluted fractions. The next day, the cells were treated with 2 μg/ml of DNT, and stained with anti-DNT polyclonal antibody as described in Methods. Bar, 5 μm. **(C) **Each fraction from the chromatography was subjected to SDS-PAGE followed by silver staining. The arrows and arrowheads indicate the proteins identified by mass spectrometry. The asterisk indicates contaminated human keratin. (**D**) The fractions from chromatography with the culture supernatant of MC3T3-E1 cells. Nidogen-2 was detected at approximately 200 kDa, and the smaller variants of nidogen-2 are presumed to be N-terminally truncated, based on the results of mass spectrometry (arrowheads). Note that the band indicated by open arrowhead is present in fraction 4 inducing the association of DNT with the FN network.

**Figure 6:**
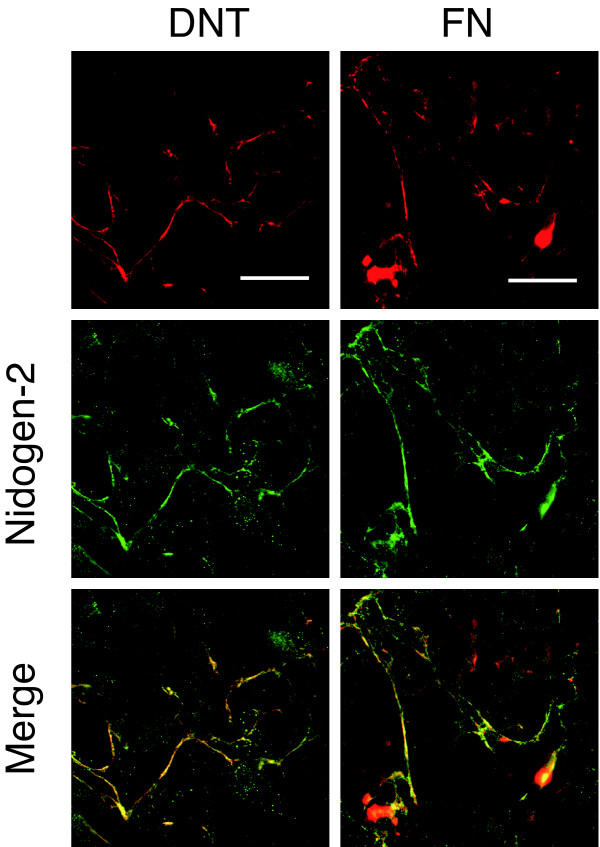
**Colocalization of nidogen-2 and DNT or FN**. MC3T3-E1 cells incubated with DNT were stained with anti-nidogen-2 polyclonal antibody, and anti-DNT or anti-FN monoclonal antibodies. Bars, 5 μm.

We tried to isolate the unknown component from the culture supernatant of FN-null cells by ion-exchange chromatography (Fig. 5A). Each fraction eluted by Mono Q anion-exchange chromatography was added to the culture of MRC-5 cells, and checked for the ability to recruit DNT to the FN network. Simultaneously, each fraction was subjected to SDS-PAGE and proteins in the fractions were identified by mass spectrometry. Fraction 4 apparently induced the association of DNT with the FN network on MRC-5 cells (Fig. 5B). Mass spectrometry revealed that fraction 4 contains ECM-related proteins such as nidogen-2 in an N-terminally truncated form (open arrowhead), and lysyl oxidase-homolog 2 (LOXL2) and 3 (LOXL3) (Fig. 5C). Similar results were obtained from the culture supernatant of MC3T3-E1 cells: the truncated form of nidogen-2 (open arrowhead) and LOXL3 were found in fraction 4, which induced the association of DNT with the FN network on MRC-5 cells (Fig. 5D). LOXL2 was expressed at neither the mRNA nor protein level in MC3T3-E1 cells, which show intensive colocalization of DNT and the FN network (Fig. 3). LOXL3 supplemented to the culture did not induce the colocalization of DNT with the FN network on MRC-5 cell (data not shown). Therefore, the truncated form of nidogen-2 but not LOXL2 or 3 is considered to be a possible candidate for the unknown intermediate between DNT and FN. Nidogen-2 was found to organize a network on the cells and colocalize with DNT and FN (Fig. 6).

### DNT is liberated from the FN network and affects sensitive cells

**Figure 7:**
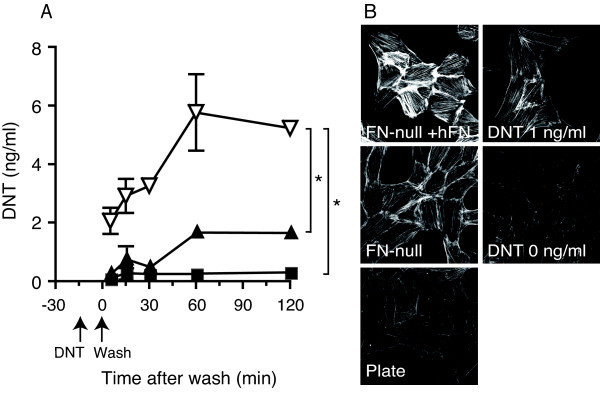
**DNT associated with the FN network diffuses from the cell surface and affects sensitive cells**. **(A) **The concentration of DNT diffused from FN-null cells supplemented with hFN (open triangles) or not (closed triangles). The culture supernatant of the cells was obtained as described in Methods, and the DNT concentration was determined. As a control, the medium incubated without FN-null cells (closed squares) was prepared in the same manner. The abscissa indicates the time after the washing of DNT-treated cells. Each plot represents the mean ± S.D. (n = 3). Asterisks indicate significant differences (*P *< 0.001). **(B) **Stress fiber-inducing activity of DNT liberated into the culture supernatant. The culture supernatant was obtained 15 min after washing in the experiments of panel A, and examined for DNT activity on MC3T3-E1 cells. Actin fibers were visualized by rhodamine-phalloidin. The left panels show MC3T3-E1 cells incubated with each culture supernatant and the right panels show the cells incubated with DNT. The experiments were performed three times and representative results are shown. Bar, 5 μm.

We examined whether DNT liberated from the FN network was still active (Fig. 7). FN-null cells supplemented with or without human FN were treated with DNT, and the amount of toxin that diffused from the cells after replacement of the medium was measured by ELISA. DNT gradually diffused from the FN-supplemented FN-null cells in 60 min (Fig. 7A). Its concentration was about three times that which diffused from unsupplemented cells. The diffused toxin caused the reorganization of actin stress fibers in MC3T3-E1 cells, indicating that it was still active even after its association with, and liberation from, the FN network (Fig. 7B).

## Discussion

Here, we found that DNT temporarily associated with the FN network on cells. FN, a major component of the ECM, is mainly produced by fibroblasts and organized into a fibrillar network through binding to cell surface receptors, integrins [14-16]. A DNT mutant deficient in transglutaminase activity was also associated with the FN network (data not shown), indicating that the enzymatic activity of DNT is not required for the association. Because deletion mutants of DNT, in which any of the regions is missing, and heat-inactivated DNT did not associated with the FN network (data not shown), the overall structure of the toxin may be crucial to the association. DNT did not colocalize with the FN network generated by MRC-5 cells, suggesting that it interacts with FN not directly, but via another cellular component. Nidogen-2 in an N-terminally truncated could be a candidate for the component, because it was present in only the fraction which induced the association of DNT with the FN network on MRC-5 cells, whereas full-length nidogen-2 did not. Although its biological importance is not fully understood, nidogen-2 is known to interact with various molecules in the ECM [17]. The nature of the truncated nidogen-2 is currently unknown. How the truncated nidogen-2 mediates the association between DNT and the FN network is not known either. At least, we observed that nidogen-2 was colocalized with not only FN but also DNT in the fibrillar structure. SBED-DNT crosslinked to two distinct components in addition to FN (Fig. 1C). These two components might be other candidates to intermediate the association between DNT and the FN network. However, they could not be isolated by combinations of anion- and cation-exchange chromatographies, probably because of their instability. In addition, the living cells, some cell membrane proteins, and/or the fibrillar structure of FN may be also required, because we could not reproduce the association of DNT with FN in the presence of the culture supernatant of FN-null cells by *in vitro *techniques such as ELISA and immunoprecipitation (data not shown). DNT may associate with the FN network by a complicated mechanism involving the truncated nidogen-2 and other cellular components. We are now conducting further work to elucidate this issue.

The association of DNT with the FN network was seen in not only DNT-sensitive cells but also insensitive cells, which indicates that the FN network neither serves as a receptor for the toxin nor is involved in the intoxicating procedures of the toxin on sensitive cells. Instead, the FN network may serve as a transient storage site for DNT, allowing the toxin to reach target cells or tissues more efficiently. Components of the ECM including FN are known to bind and regulate various growth factors such as insulin-like growth factor (IGF), fibroblast growth factor (FGF), transforming growth factor-beta (TGF-β), hepatocyte growth factor (HGF), and vascular endothelial growth factor (VEGF) [18,19]. These growth factors are released from the ECM in response to alterations in the extracellular environment and exert biological effects to regulate cell survival, proliferation, and differentiation. For example, VEGF is associated with the ECM via FN or heparan sulfate at acidic pH. When the pH of the extracellular milieu increases, VEGF is released from the ECM network and activates its functional receptor to induce angiogenesis [20,21]. This pH-dependent association of VEGF is considered a key mechanism determining the direction of newly developed blood vessels in wound healing and tumor metastasis. The association of DNT with the FN network was also dependent on the pH of the extracellular environment. *Bordetella *infections are reported to be accompanied by necrosis or the desquamation of superficial epithelial layers with inflammatory responses [22,23]. These events may facilitate the exposure of newly generated ECM containing FN. The inflammatory locus is reportedly characterized by local acidosis due to lactic acid production [24]. FN is actively produced by fibroblasts and osteoblasts, mesenchymal cells, which could be targets for DNT. Therefore, it is conceivable that DNT binds to the ECM containing FN at low pH in inflammatory areas during an infection, and by repeatedly associating with and diffusing from the FN network, moves deep into tissue where the density of FN should be higher, eventually reaching target cells. This may explain how DNT, which is not secreted by bacteria and is present at low concentrations in extrabacterial milieus, can affect target tissues in *Bordetella *infections such as atrophic rhinitis.

## Conclusions

DNT associates temporarily with FN-based ECM network. The association seems to be mediated by the truncated-form of nidogen-2 and/or some cellular components, which have an affinity to the FN network. It is likely that the FN network does not function as a specific receptor but serves as a temporary storage system for DNT, enabling the small amount of the toxin to effectively reach target cells across the epithelia and connective tissue.

## Methods

### Cell culture

Mouse preosteoblastic cells MC3T3-E1 were cultured in alpha modified Eagle's medium (α-MEM) supplemented with 10% fetal calf serum (FCS). Mouse embryonic fibroblasts Balb3T3 and human fibroblasts MRC-5 were cultured in Dulbecco's modified Eagle's medium (DMEM) supplemented with 10% FCS. FN-null cells, which were kindly provided by Dr. Sottile [25], were maintained in an 1:1 mixture of Cellgro (Mediatech) and Aim V (GIBCO/Invitrogen).

### Reagents and antibodies

Human plasma FN was purchased from Sigma. Collagen to coat culture plates and slide glasses was from Nitta gelatin Co. (Cellmatrix, Osaka, Japan). The monoclonal (FN-15, F7387) and polyclonal (F3648) antibodies against FN and polyclonal antibody against laminin (L9393) were obtained from Sigma. The anti-mouse nidogen-2 (M-300, sc-33143) and anti-collagen type I (234168) antibodies were from Santa Cruz and Calbiochem, respectively. The anti-DNT monoclonal antibody 2B3 and anti-DNT polyclonal antibody were prepared as reported [4,26]. Alexa 488-conjugated goat anti-rabbit IgG, Alexa 546-conjugated goat anti-mouse IgG, and Alexa 488-conjugated streptavidin were from Molecular Probes/Invitrogen. Horseradish peroxidase (HRP)-conjugated streptavidin was from Chemicon. DNT that is N-terminally fused with hexahistidine was obtained as reported [27].

Sulfo-SBED, a trifunctional cross-linking reagent, was purchased from Thermo scientific. 5-carboxyfluorescein, succinimidyl ester (5-FAM, SE) was obtained from Molecular Probes/Invitrogen. For conjugation, DNT was dialyzed against 0.1 M NaHCO_3_, pH 8.3, mixed with Sulfo-SBED or 5-FAM, SE at a molar ratio of 1:32, and incubated at room temperature for 30 min. After incubation, the unconjugated reagent was removed by gel filtration with a PD-10 column (GE Healthcare).

### Immunofluorescent staining of DNT-treated cells

MC3T3-E1, Balb3T3, and MRC-5 cells were seeded at 50,000 cells/cm^2 ^in wells of a 24-well plate with cover glasses and grown overnight. FN-null cells were cultured overnight on collagen-coated cover glasses in Cellgro-Aim V with or without 10 μg/ml of human FN. The next day, the medium was replaced with a fresh batch containing 2 μg/ml of DNT, 5-FAM-conjugated DNT (5-FAM-DNT) or SBED-conjugated DNT (SBED-DNT), and the cells were incubated for 15 min at 37°C. The cells were then fixed with 3% paraformaldehyde in Dulbecco's modified phosphate-buffered saline (D-PBS (-)) for 10 min and treated with primary antibodies for 1 h, and subsequently secondary antibodies for 30 min in the presence of 10% FCS. The cells were washed three times with D-PBS (-) after each procedure. The cells were mounted in Fluoromount (Diagnostic BioSystems) and imaged with an OLYMPUS BX50 microscope controlled by SlideBook 4.0 (Intelligent Imaging Innovation, Inc.).

Anti-DNT polyclonal or monoclonal antibodies were used at 10 μg/ml for DNT staining. FN, collagen typeI, laminin, and nidogen-2 were stained with the respective antibodies at concentrations indicated in the instruction manuals.

### Cross-linking of MC3T3-E1 cells with SBED-conjugated DNT

Confluent MC3T3-E1 cells in a 10-cm dish were treated with 2.5 μg/ml of SBED-DNT at 37°C for 15 min and then exposed to UV light at 365 nm for 5 min. The cells were washed with D-PBS (-) twice and solubilized with D-PBS (-) containing 1% NP-40 and 1% protease inhibitor cocktail (Nacalai, Kyoto, Japan) at 4°C for 60 min. The solubilized cells were centrifuged at 10,000 × g for 20 min at 4°C and the supernatant and pellet were collected as the soluble fraction and insoluble fraction, respectively. The insoluble fraction was sonicated in D-PBS (-) containing 5 μg/ml of DNase I and 8 M urea. After centrifugation, the supernatant was injected into a Mini Q column (0.32 × 3 cm, GE Healthcare), and eluted with a gradient of 0-1 M NaCl in 20 mM Tris-HCl (pH 8.5), containing 8 M urea, using the SMART system (GE Healthcare).

### Screening for components intermediating the association between DNT and the FN network

FN-null cells or MC3T3-E1 cells were cultured in FCS-free DMEM or α-MEM for 72 h. The supernatant of the culture was dialyzed against 20 mM Tris-HCl, pH 8.5 containing 0.5 M NaCl, and subjected to anion-exchange chromatography with a HiTrap Q column (0.7 × 2.5 cm, GE Healthcare). The materials absorbed to the column were eluted in 1-ml fractions with a linear gradient of 0.5-1 M NaCl, and each fraction was tested for the ability to recruit DNT to the fibrillar structure on MRC-5 cells using immunofluorescence microscopy. The positive fractions were collected, appropriately diluted, and mixed with 5% CHAPS and 10 M urea to make a solution of 20 mM Tris-HCl, pH 8.5, containing 50 mM NaCl, 0.5% CHAPS and 6 M urea. The sample was subjected to Mono Q anion-exchange chromatography, and eluted with a linear gradient of 0.05-1 M NaCl. The eluted fractions were examined again for the ability to recruit DNT to the fibrillar structure on MRC-5 cells. Proteins contained in the positive fraction were identified by mass spectrometry as mentioned below.

### DNT diffusion assay

FN-null cells were seeded in wells of a 24-well plate at 25,000 cells/cm^2 ^and grown overnight. The next day, the cells were washed well with Cellgro-Aim V and incubated overnight in the same medium with or without 10 μg/ml of human FN. The culture medium was replaced with a fresh batch containing 2.5 μg/ml of DNT and the cells were incubated for 15 min at 37°C. After three washes with FCS-free DMEM, the cells were further incubated in the fresh medium. The culture supernatant was taken at the indicated time point, and an aliquot was applied to MC3T3-E1 cells without dilution. After incubation at 37°C overnight, the cells were examined for actin stress fibers as described previously [27]. Another aliquot of the culture supernatant was examined for DNT by sandwich-ELISA, performed with a 96-well plate coated with anti-DNT polyclonal antibody. After blocking with 0.2% BSA at 4°C overnight, each sample was added to the plate in triplicate and incubated for 2 h at 37°C. The plate was treated with biotin-labeled anti-DNT antibody, followed by HRP-conjugated streptavidin for 1 h at 37°C. BM Blue POD substrate (Roche) was used as an HRP substrate and the reaction was stopped by 1 M H_2_SO_4_. The wells were washed four times with D-PBS (-) containing 0.05% Tween-20 between each step. The concentration of DNT was estimated from a standard curve made with a DNT preparation.

### Other methods

Protein concentrations were determined using BCA Protein Assay Reagents (Thermo Scientific). For the mass spectrometric analysis, samples were subjected to SDS-PAGE, followed by staining using a Silver stain MS kit (Wako). Visualized proteins were exercised from the gels, and digested with trypsin according to a method described elsewhere [28]. Mass spectrometric data were analyzed with the MASCOT program (Matrix Science Ltd.). The statistical differences among groups of data were analyzed by one-way analysis of variance (ANOVA), followed by a Bonferroni posttest, using GraphPad Prism software version 4 (GraphPad Software, Inc.)

## Abbreviations

(DNT): Dermonecrotic toxin; (ECM): extracellular matrix; (FN): fibronectin

## Authors' contributions

AF-M participated in the design and performed all experiments and drafted the manuscript. SK and MM contributed to the interpretation of data. YH obtained funding for designed the research and critically revised the manuscript. All authors read and approved the final manuscript.
